# Increased Intrathecal High-Avidity Anti-Tau Antibodies in Patients with Multiple Sclerosis

**DOI:** 10.1371/journal.pone.0027476

**Published:** 2011-11-29

**Authors:** Lenka Fialová, Ales Bartos, Jana Švarcová, Ivan Malbohan

**Affiliations:** 1 Institute of Medical Biochemistry, First Faculty of Medicine, Charles University in Prague, Prague, Czech Republic; 2 Department of Neurology, Third Faculty of Medicine, University Hospital Královské Vinohrady, Charles University in Prague, Prague, Czech Republic; 3 Prague Psychiatric Center, Prague, Czech Republic; 4 Institute of Clinical Biochemistry and Laboratory Diagnostics, First Faculty of Medicine, General Teaching Hospital, Charles University in Prague, Prague, Czech Republic; Institute Biomedical Research August Pi Sunyer (IDIBAPS) - Hospital Clinic of Barcelona, Spain

## Abstract

**Background:**

Antibodies against tau protein indicate an interaction between the immune system and the neurocytoskeleton and therefore may reflect axonal injury in multiple sclerosis (MS).

**Methodology/Principal Findings:**

The levels and avidities of anti-tau IgG antibodies were measured using ELISA in paired cerebrospinal fluid (CSF) and serum samples obtained from 49 MS patients and 47 controls. Anti-tau antibodies were significantly elevated intrathecally (p<0.0001) in the MS group. The CSF anti-tau antibody levels were lower in MS patients receiving therapy than those without treatment (p<0.05). The avidities of anti-tau antibodies were higher in the CSF than in the serum (MS group p<0.0001; controls p<0.005). Anti-tau avidities in the CSF were elevated in MS patients in comparison with controls (p<0.05), but not in serum.

**Conclusions:**

MS patients have higher levels of intrathecal anti-tau antibodies. Anti-tau antibodies have different avidities in different compartments with the highest values in the CSF of MS patients.

## Introduction

Demyelination and axonal pathology are the main underlying processes in multiple sclerosis (MS) [1]. Axonal damage in MS has been well documented using histopathological studies, which reported axonal swelling and bulb formation [2]. Morphological changes can be reflected in biochemical findings involving an elevation of some cytoskeletal structures in the cerebrospinal fluid (CSF) of MS patients [3], [4], [5], [6].

The targeted axonal antigens for anti-neurocytoskeletal antibody responses in MS have already been investigated. Several studies reported the presence of antibodies against axonal antigens in the serum and CSF of patients having a variety of neurological diseases [7], [8], [9], [10], [11]. Previously we investigated autoantibodies to neurocytoskeletal structures such as light and medium subunits of neurofilaments and tubulins [12], [13], [14]. We found elevated intrathecal synthesis of antibodies to medium neurofilaments and higher levels of anti-tubulin antibodies in the CSF in MS patients.

In this study we were interested to see if there was a similar pattern of anti-neurocytoskeletal response against tau protein, a low-molecular-weight microtubule associated protein abundantly present in the central nervous system. It is primarly expressed in a body of the cell and then it is localized in axons [15], [16]. It involves in the axonal transport by preventing or slowing microtubule depolymerization [17]. Several studies have described that tau protein can be released into the extracellular fluid and leak into the cerebrospinal fluid during the process of axonal damage in the same way as other neurocytoskeletal structures [3], [5]. Serum anti-tau antibodies both IgG isotype and IgM are present in Alzheimer's disease patients as well as in healthy controls. The higher serum anti-phosphorylated tau antibodies of IgM isotype was observed in patients with Alzheimer's disease in comparison with controls [18]. It seems that these naturally occurring antibodies may be associated with the autoimmune process against tau proteins [18].

We also investigated the biological activity of anti-tau antibodies in more details. It is known that the antibodies are heterogeneous relative to types, classes, and avidities. Since antibody levels may not be the only feature for characterization, we also assessed avidities of anti-tau antibodies in the serum and CSF of MS patients. We hypothesized that the levels and the avidities of anti-tau antibodies would be increased in MS patients compared to neurological controls. We also wanted to determine if anti-tau antibodies and their avidities could provide some insight regarding therapeutic effects. Furthermore, we studied the relationship between levels of anti-tau antibodies and their avidities in serum and in CSF separately and also between these fluids.

## Materials and Methods

### Ethics

All subjects gave written informed consents regarding study participation. The Ethics Committee of the Third Faculty of Medicine, Charles University, Prague approved the study.

### Participants

Paired CSF and serum samples were obtained from 49 MS patients and 47 patients with other neurological diseases. The clinical data about patients and the type of therapy for MS patients are presented in Table 1.

**Table 1:** Basic clinical characteristics of multiple sclerosis patients and control subjects.

Diagnostic group	All MS	CIS	RR-aMS	RR-rMS	SPMS	PPMS	Controls
Number of patients	49	8	18	11	9	3	47
Female sex N (%)	31 (63)	5 (63)	13 (72)	4 (37)	8 (89)	1 (33)	27 (57)
Age at LP (years)	38 (27–41)	29 (27–36)	29 (26–36)	37 (27–44)	41 (38–50)	46 (38–47)	35 (30–48)
Disease duration until LP (years)	2.5 (0.5–6.5)	0.1 (0–0.55)	4.5 (1,0–5,0)	1.0 (0.5–2.0)	7.0 (6.0–11.0)	3.0 (1.0–20.0)	na
EDSS at LP	2.5 (1.0–3.5)	1.0 (0.5–1.0)	2.5 (2.0–3.0)	1.0 (0–2.0)	4.5 (4.0–5.0)	3.0 (3.0–3.5)	na
Therapy							
none	23	5	9	7	0	2	na
IS alone or with IM	26	3	9	4	9	1	na
IgG index	0.64 (0.56–0.88)	0.60 (0.61–1.01)	0.67 (0.61–1.0)	0.68 (0.6–0.93	0.73 (0.55–0.87	0.62 (0.51–1.1)	0.49 (0.45–0.53)

Data are expressed as number or median (25^th^–75^th^ percentile).

N = number of patients; MS = multiple sclerosis; CIS = clinically isolated syndrome; RR-a = relapsing-remitting form during attack; RR-r = relapsing-remitting form during remission; SP = secondary progressive form; PP = primary progressive form; EDSS = Expanded Disability Status Scale; LP = lumbar puncture; IS = immunosuppressive therapy (corticosteroids or azathioprine or both); IM = immunomodulator therapy (interferon-beta or glatiramer acetate); na = not applicable.

The IgG index indicating intrathecal production of total IgG was calculated as the CSF/serum ratio of concentration of IgG related to the albumin CSF/serum ratio. Pathological values are above 0.7.

The diagnosis and the course of MS, evaluated at the time of lumbar puncture (LP), were determined using established criteria [19], [20]. Twenty-nine patients were classified as having the relapsing-remitting (RR) form of MS, either during an attack (RR-aMS) (18 patients) or during remission (RR-rMS) (11 patients). Nine patients had the secondary progressive (SPMS) form of MS and three patients were classified as having the primary progressive (PPMS) form. Eight patients with a first acute neurological event and magnetic resonance imaging findings suggestive of multiple sclerosis were diagnosed to have a clinically isolated syndrome (CIS) at the time of lumbar puncture [20]. The disability score for all MS patients was judged using the Expanded Disability Status Scale (EDSS) [21].

Twenty-three patients had not received any therapy prior to their lumbar puncture, twenty-six patients had been treated with immunosuppressive (IS) drugs (steroids or azathioprine or both) either alone or in combination with immunomodulators (IM) (interferon-beta or glatiramer acetate).

Controls were recruited from neurological patients with heterogeneous diseases. This group consisted of individuals without obvious neuro-axonal disease (e.g. cephalea), i.e. who were suspected to have neurological disease and thus received lumbar puncture but finally turned out not to be affected. It also comprised subjects mainly with polyneuropathy and cranial neuropathy. Twelve patients had aseptic neuroinfection. Except for the patients with neuroinfection we assumed the rest of the controls as normal in terms of antibody status. It is difficult or almost impossible due to ethical reasons to obtain cerebrospinal fluid samples from healthy people, people without neurological disease and with other inflammatory non-neurological diseases.

Specimens were stored at −20°C until analyzed.

### Methods

#### ELISA determination of anti-tau IgG antibodies and their avidities

CSF and serum IgG anti-tau antibodies were analyzed using newly developed ELISA techniques based on the ELISA method described by Silber et. al. [8] and our prior experience [12], [13]. Avidities were determined using ELISA methods described by Matheus et al. [22] with additional specific modifications required for anti-tau antibodies. Urea, a mild denaturant, was used to disrupt immune complexes.

Tau protein is a highly conserved protein and the amino acid sequences of bovine and human tau are highly homologous [23]. The human tau is commercially available only as a recombinant form, in which the post-translational modifications (for example phosphorylation and glycosylation) are lacking. The immunoreactivity of human recombinant tau protein can be changed. Due to both reasons we used commercially available bovine tau protein (Cytoskeleton, USA) as the antigen for coating of the microplate wells.

For the avidity determination, an extra step using a reducing agent (urea) was added; this step facilitated disruption of antigen-antibody binding. Usually the 6 M or 8 M urea solution is used in avidity experiments [22], [24], [25]. Our preliminary experiments showed that 8 M urea solution disrupted the bindings in immunocomplexes of tau protein and anti-tau antibodies in the wells most effectively. Therefore we used 8 M urea as recommended in the method described by Matheus et al. [22].

The procedure for estimating anti-neurocytoskeletal antibodies to tau proteins is as follows. Individual 96-well microplates (Maxisorp, NUNC, Roskilde, Denmark) were coated with 50 µL of bovine tau protein (2.5 µg/ml) in bicarbonate coating buffer (pH = 9.6). The microplates were then allowed to incubate overnight at 4°C. Blocking with 1% bovine serum albumin (BSA) in phosphate buffered saline (pH = 7.2) was performed for 1 hour at room temperature. After washing, 50 µL of sera, diluted 1∶400 in 1% BSA in PBS or undiluted CSF, was added to the wells in quadruplicate and incubated for 2 hours at 37°C. The plates were then washed three times. One hundred microliters, of 8 mol/L urea solution, was added to half of the wells and 100 µl of PBS was added to the other half of the wells. After a 10 min incubation, at 37°C, the wells were washed with wash buffer. In the next step horseradish peroxidase-conjugated goat anti-human IgG (Sevapharma, Prague, CZ), diluted 1∶5000 in bovine serum solution in PBS, was added to all appropriate wells and the plates were incubated for 90 minutes at 37°C. After incubation the plates were washed. Color was developed by adding substrate (tetramethylbenzidine with H_2_O_2_) (TEST-LINE, Brno, CZ) for 30 minutes at room temperature. The enzymatic reaction was stopped by adding 2 mol/L H_2_SO_4_. The absorbance of each well was read at 450 nm, with a reference filter of 620 nm, using a microplate reader (Labsystem, Finland).

Anti-tau antibodies were examined in all patients of both groups. The avidity was determined in 38 MS patients and in 25 age- and sex-matched control subjects.

#### Calculations of results

The same pool of human sera was used as a standard in all of the analytical series for comparative purposes. A standard curve was generated from the serial dilutions of the stock pooled serum. A best fit curve through the points on the graph was drawn. The absorbances were transformed into arbitrary concentration units using a standard curve. Serum concentrations were multiplied by a dilution factor (400×).

Avidity results were expressed as an avidity index. The index represents the percentage of residual auto-antibodies bound in the wells, in the presence of urea solution, to the total auto-antibody bound in the absence of urea. It was calculated by dividing absorbance (or arbitrary units) values obtained from the avidity ELISA assay (with urea addition) by absorbance (or arbitrary units) values obtained using the standard ELISA method (without urea) and multiplying by 100. There was a strong correlation between these two ways of calculations (Spearman correlation coefficient r = 0.95, p<0.0001). In this study we used absorbation for the calculation of avidity.

Intrathecal synthesis of anti-tau IgG antibodies was estimated as an anti-tau antibody index. The following formula was used: [(CSF anti-tau/serum anti-tau)/(CSF albumin/serum albumin)].

#### Determination of albumin and total IgG in serum and cerebrospinal fluid

The concentrations of albumin and total IgG in serum and cerebrospinal fluid were assayed using immunonephelometry.

### Statistical methods

Since data were not normally distributed and the number of patients in MS subgroups was low, differences among all groups were analyzed using the Kruskal-Wallis test followed by the Mann-Whitney U test. The Wilcoxon pair test was used for statistical analysis of avidity differences in the serum and CSF. The Spearman's coefficient was used for correlation analyses. The significance level for all tests was p<0.05. The Bonferroni correction was applied for multiple comparisons. Statistical analysis was performed using Statistica 8 software (StatSoft, Inc. Tulsa, OK, USA).

## Results

The basic clinical and laboratory variables of the MS patients and the control group are shown in Table 1. There was no difference in age and sex between MS patients and controls. The IgG indices in the MS group were higher than those in the controls (p<0.0001).

### Anti-tau antibodies in the serum and CSF and their intrathecal synthesis

**Figure 1:**
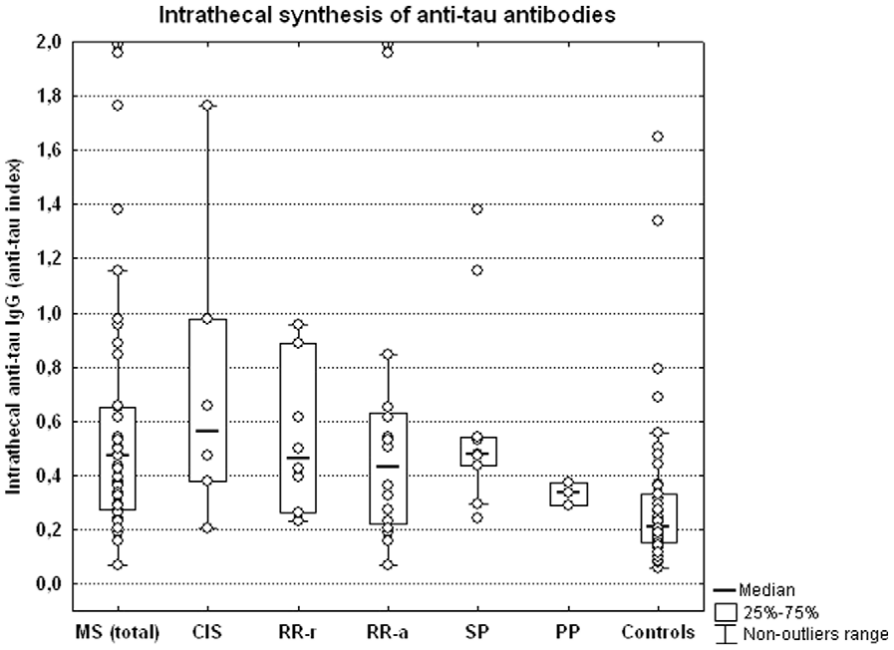
Intrathecal synthesis of anti-tau antibodies. The intrathecally synthesized anti-tau IgG antibodies were significantly higher in the MS group as a whole than those in the controls (p<0.0001). There were significant differences in intrathecal synthesis of anti-tau antibodies between CIS, RR-rMS, RR-aMS or SP forms of MS and the control group (CIS vs. controls: p<0.01; RR-rMS vs. controls: p<0.005; RR-aMS vs. controls: p<0.05; SPMS vs. controls: p<0.005). Abbreviations: MS = multiple sclerosis; CIS = clinically isolated syndrome; RR-a = relapsing-remitting form during attack; RR-r = relapsing-remitting form during remission; SP = secondary progressive form; PP = primary progressive form; Ig = immunoglobulin.

The serum and CSF anti-tau IgG antibodies levels tended to be higher in MS patients (as a group) than in controls, but the elevation did not reach statistical significance. Intrathecal synthesis of anti-tau antibodies was significantly elevated in the MS group compared with controls (p<0.0001) (Fig. 1). The levels of anti-tau antibodies were about 500 times higher in serum than in the CSF.

In a more detailed analysis of the MS subgroups, based on clinical course, we observed similar serum and CSF anti-tau antibody levels in various individual clinical forms of MS as well as in controls. Intrathecal synthesis of anti-tau antibodies was significantly higher in all MS clinical forms than in the control group (CIS vs. controls: p<0.01; RR-rMS vs. controls: p<0.005; RR-aMS vs. controls: p<0.05; SPMS vs. controls: p<0.005), but a large overlap among different groups was observed. Analysis of PPMS was not performed because of small group size (3 patients only) (Fig. 1).

### Anti-tau avidities in the serum and CSF

**Figure 2:**
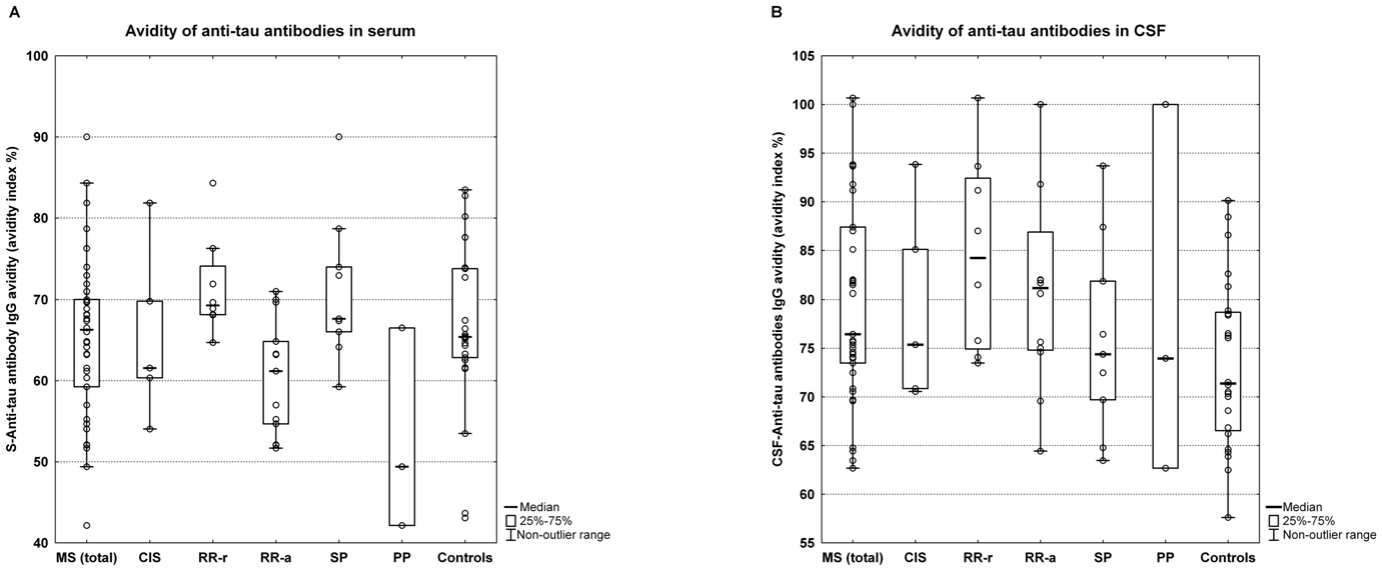
Avidities of CSF and serum anti-tau antibodies. A. Avidity of anti-tau antibodies in serum. There was no difference between the totals MS patients and controls. The patients in RR-rMS and SPMS subgroups had significantly higher serum anti-tau avidities in comparison with RR-aMS (RR-rMS vs. RR-aMS: p<0.01; SPMS vs. RRaMS: p<0.01). The serum avidities in RR-aMS were slightly lower than those in the control group (p<0.05). B. Avidity of anti-tau antibodies in CSF. Avidities of anti-tau IgG antibodies in CSF were significantly higher in the MS group as a whole than in the controls (p<0.05). There was also a significant difference in avidities between RR-rMS subgroup and the control group (p<0.05). The avidities of anti-tau antibodies in the CSF were significantly elevated in comparison with those in the serum in both the MS group and the controls (MS group: CSF vs. serum p<0. 0001; controls: CSF vs. serum p<0. 005). Compare corresponding box plots – MS (total) and controls between Fig. 2A and 2B. Abbreviations: CSF = cerebrospinal fluid; S = serum; other abbreviations are explained in the Fig. 1.

Serum anti-tau avidities did not vary between total MS patients and controls. However, further analysis among the MS forms showed that patients in RR-rMS and SPMS subgroups had significantly higher serum anti-tau avidities compared with RR-aMS (RR-rMS vs. RR-aMS: p<0.01; SPMS vs. RR-aMS: p<0.01) (Fig. 2A). The serum avidities in RR-aMS were even lower than those in the control group (p<0.05).

On the other hand, CSF anti-tau avidities were significantly elevated in MS patients compared with controls (p<0.05). There was also a significant difference between the RR-rMS subgroup and the control group (p<0.05) (Fig. 2B).

The avidities of anti-tau antibodies were significantly higher in the CSF in comparison with those in the serum in both the MS group and the control group (MS group: CSF vs. serum p<0.0001; controls: CSF vs. serum p<0.005).

### Influence of therapy on the anti-tau antibody IgG levels and avidities

Additionally, we compared the anti-tau antibody levels and their avidities in the treated patients and untreated ones. Patients were divided into two subgroups – patients untreated prior to their LP and those treated with immunosuppressive therapy alone or in combination with immunomodulators at the time of their LP. The treated patients had significantly higher the EDSS score in comparison with the untreated group (p<0.0001).

**Figure 3:**
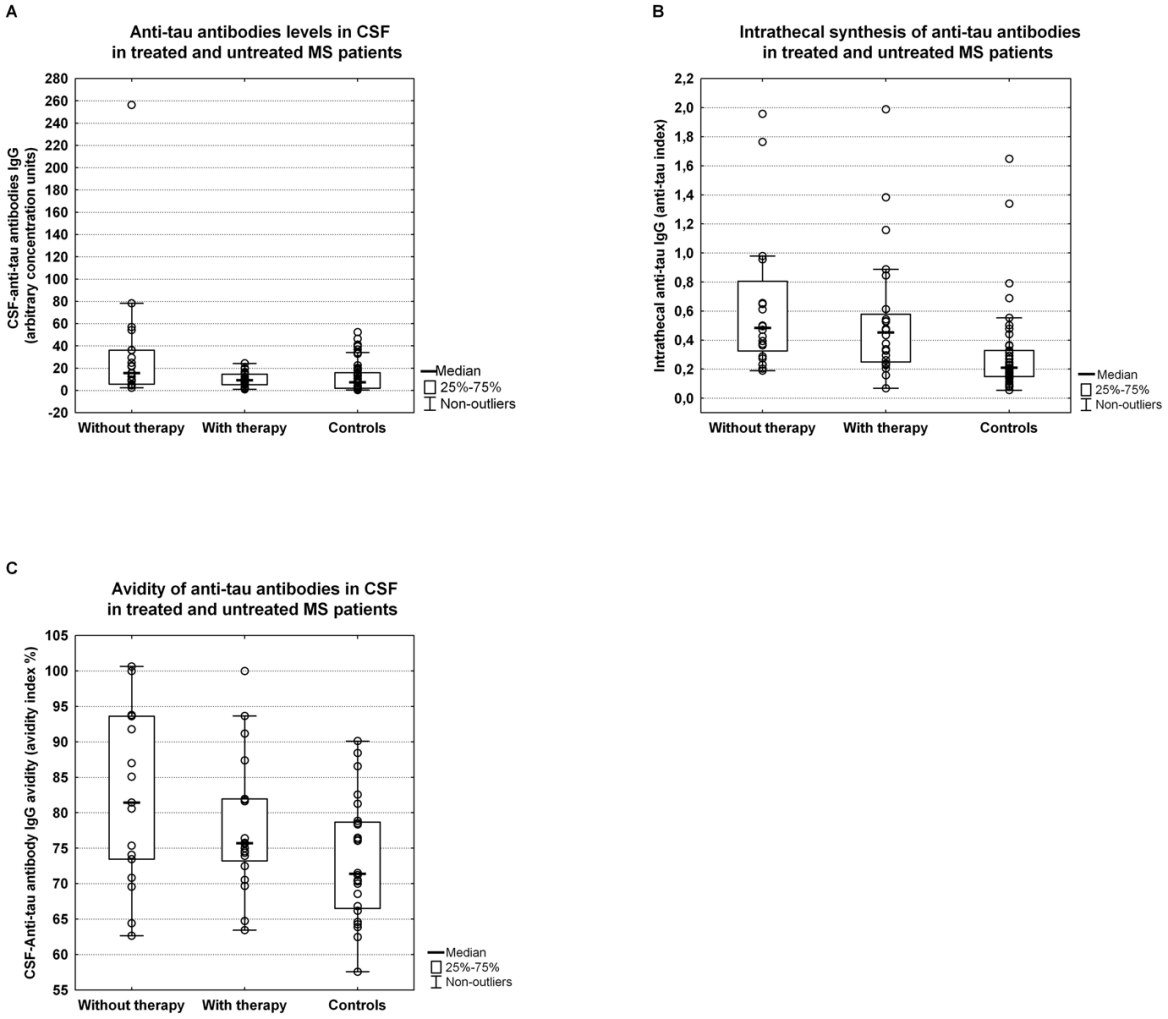
Relationship between therapy and anti-tau antibody levels. A. Anti-tau antibodies levels in CSF in treated and untreated MS patients. There were differences between the subgroup of patients with therapy and those without therapy (p<0.05) or between the control group and patients without therapy (p<0.01) in the CSF anti-tau levels. B. Intrathecal synthesis of anti-tau antibodies in treated and untreated MS patients. The treated patients and untreated ones had significantly elevated intrathecal synthesis of anti-tau antibodies compared to those in the control group (treated patients vs. controls p<0.001; untreated patients vs. controls p<0.001). C. Avidity of anti-tau antibodies in CSF in treated and untreated MS patients. CSF anti-tau avidities were significantly higher in MS patients without therapy than the controls (p<0.005). There were no differences between treated MS patients and untreated MS ones or controls. Levels and avidities of serum anti-tau antibodies did not differ between the MS subgroups and controls (figure not shown). Abbreviations are explained in the Fig. 1 and 2.

Serum anti-tau antibodies were suppressed but without statistical significance in the subgroup of MS patients receiving therapy. With regard to the CSF, MS therapy resulted in a significant decrease in CSF anti-tau antibodies. Untreated MS patients had significantly higher levels of CSF anti-tau antibodies than patients receiving therapy (p<0.05) or the control group (p<0.01) (Fig. 3A). On the other hand, therapy did not significantly affect intrathecal synthesis of anti-tau antibodies. Both subgroups of MS patients (treated patients and untreated ones) had significantly elevated intrathecal synthesis of anti-tau antibodies compared to control patients (treated patients vs. controls p<0.001; untreated patients vs. controls p<0.001) (Fig. 3B).

There were no differences in serum anti-tau avidities between either of the MS subgroups and the controls. CSF anti-tau avidity was significantly higher in untreated MS patients than controls (p<0.005) while it did not differ between treated MS patients and untreated ones or controls (Fig. 3C).

### Correlations

#### Relationships of antibody levels between and in CSF and serum

A highly significant correlation between CSF and serum anti-tau IgG antibodies was observed in the MS group as well as in the control group (r = 0.6, p<0.0001; r = 0.7, p<0.0001; respectively). There were three outliners in the MS group. We performed the same analysis without them and we received similar results.

Specific CSF anti-tau IgG significantly correlated with total CSF IgG (MS group: r = 0.5, p<0.001) in the MS groups. A similar relationship in serum was also found for specific anti-tau IgG and total IgG (MS group: r = 0.5, p<0.001; controls: r = 0.3, p<0.05) (Table 2).

**Table 2:** Relationships of anti-tau antibody levels between and in CSF and serum.

Group	Correlations	n	r	p
MS group	S-anti-tau×CSF-anti-tau	49	**0.6**	**<0.0001**
	S-anti-tau×S-IgG total	49	**0.5**	**<0.001**
	CSF-anti-tau×CSF-IgG total	45	**0.5**	**<0.001**
Controls	S-anti-tau×CSF-anti-tau	46	**0.7**	**<0.0001**
	S-anti-tau×S-IgG total	47	**0.3**	**<0.05**
	CSF-anti-tau×CSF-IgG total	43	0.3	n.s.

Abbreviations.

MS = multiple sclerosis; S = serum; CSF = cerebrospinal fluid; n.s. = not significant; anti-tau = anti-tau antibodies; r = Spearman correlation coefficient.

#### Relationships of avidities between and in CSF and serum

Similarly to anti-tau IgG antibodies, avidities in the serum of the MS group were related to those in the CSF, but with a weaker correlation (r = 0.3, p<0.05). A relationship between total anti-tau antibodies and their avidities in the serum and CSF was not found in any group with the exception of an inverse correlation between total CSF anti-tau antibodies and their avidities in the controls (r = −0.5, p<0.05).

#### Relationships between antibody findings and clinical variables

No relationship was found between anti-tau antibodies (levels or avidities) in the serum or CSF with regard to clinical variables (age or sex of the patients) in any group. However, intrathecal synthesis was inversely related to age in MS patients (r = −0.4, p<0.01). Serum and CSF anti-tau levels and their avidities did not correlate with disease duration or patient disability, expressed as an EDSS in the MS group. No relationship between anti-tau antibodies and EDSS was observed in an additional analysis in subgroups of treated and untreated MS patients.

## Discussion

This study demonstrates, for the first time, elevated intrathecal synthesis of anti-tau IgG antibodies in patients with multiple sclerosis compared to the patients with a range of disorders from normal to inflammatory. Therefore effects measured in this study might be even larger than proposed if we took healthy individuals as controls into account. This is another indirect indication of simultaneous axonal and immune processes which were observed in our previous studies as well as studies by other researchers [8], [13].

Levels of neurocytoskeletal tau protein are increased during the early stages of MS and in those with higher intrathecal IgG synthesis [3]. This finding is in a good agreement with our observation of significantly elevated intrathecal anti-tau antibodies. Increased intrathecal synthesis of anti-tau antibodies could reflect the autoimmune humoral reaction against extracellularly released tau molecules. It seems that anti-tau antibodies might represent one of the specific IgG populations participating in an increased polyspecific humoral response. This presumption was supported by the observation of a positive correlation between specific anti-tau IgG antibodies and total IgG, expressed both in the CSF and in the serum in the MS group. The increase of intrathecal anti-tau antibodies even appeared in CIS. It reflects axonal disintegration before the development of clinically definite MS. In addition, the elevation of intrathecal anti-tau antibodies is independent of the form of MS and thus a general phenomenon typical of MS.

The absence of a difference between the anti-tau CSF antibodies in the MS and the control groups may be explained by a therapeutic impact on results. Significantly elevated levels in untreated MS patients were masked and offset by lower levels in treated MS patients.

CSF anti-tau levels were related to serum levels. Unfortunately, serum anti-tau antibodies cannot be regarded as a biomarker of axonal damage since there is no difference between MS and control groups. Similarly as Rosenmann et al. [18] in patients with Alzheimer's disease, we observed multifold higher levels of anti-tau antibodies in the serum compared to the CSF. This implies that antibody production occurs not only in the intrathecal space but also in the peripheral circulation. Released cytoskeletal antigens may pass from the CSF into the blood through a disrupted blood-CSF barrier. This may be common feature for other kinds of anti-neurocytoskeletal antibodies. In a previous study, we observed such a serum/CSF gradient for antibodies against the medium neurofilament subunit in MS patients [13].

Antibodies differ in avidities as well as levels. Antibodies in infectious and some autoimmune diseases mature from initially having low-avidity antibodies to having high-avidity antibodies later [26], [27]. Anti-tau antibodies have different avidities in different compartments (serum and CSF) with the highest values in the CSF of MS patients. Likewise, similar avidity maturation has been described for other autoimmune diseases [27]. Some studies consider high-avidity antibodies to be more pathogenic than those with low-avidity [28], [29], [30].

A surprisingly, serum anti-tau antibodies with higher avidity were present in MS remission patients, unlike those experiencing a relapse. We hypothesize that the anti-tau antibodies with higher avidity form immune complexes more easily, with appropriate antigens, and are missed during detection; immune complexes participate in a variety of pathological actions such as complement activation. Therefore, it is difficult to evaluate possible pathogenic or protective effects of high-avidity anti-tau antibodies in MS from our results.

Glucocorticoids, interferon-beta or glatiramer acetate alter B-cell functions. B-cells participate in MS processes at multiple levels [31]. We observed therapeutic effects on CSF anti-tau antibodies in lower levels without avidity change.

In contrast to our previous studies on antibodies against light and medium subunits of neurofilaments [12], [13], intrathecal synthesis of specific anti-tau antibodies did not reflect patient disability when the group of MS patients was evaluated as a whole. These results were in agreement with animal experiments in which only half of the mice immunized with the tau protein developed neurological symptoms, with no correlation between antibody titers and clinical disease development [32]. This is not surprising since axonal injury is a more complex process including not only the action of anti-neurocytoskeletal antibodies but also involving several other mechanisms [2].

Various studies addressed the role of autoantibodies in pathogenesis of some diseases [2], [32], [33], [34]. Some antibodies targeting intracellular antigen may cause neuronal damage [33], [35], [36]. Autoimmunity to another cytoskeletal protein light neurofilament induced axonal damage and neurological disease including spasticity – a common feature of MS [35]. We assume that similar underlying processes for anti-tau antibodies may occur. The autoantibodies may enter the neurons and react with their target antigens. Thus, the function of these proteins may be impaired. The precise mechanism of anti-tau antibody actions is being under investigation especially in the association with active and passive immunization with tau protein in Alzheimer disease [2], [32].

The strength of our study relates to (i) a relatively large sample size of well characterized and balanced subjects in two main groups, (ii) simultaneous measurements of levels as well as avidities of specific anti-tau antibodies both in serum and CSF using newly developed ELISA methods and (iii) study of relationship between therapy and anti-tau antibodies status. Limitations include a small number of MS patients in subgroups other than that in the RRMS subgroup, partly due to ethical reasons (SPMS) or rare occurrence in general (PPMS).

## References

[1] McFarland HF, Martin R (2007). Multiple sclerosis: a complicated picture of autoimmunity.. Nat Immunol.

[2] Huizinga R, Linington C, Amor S (2008). Resistance is futile: antineuronal autoimmunity in multiple sclerosis.. Trends Immunol.

[3] Brettschneider J, Maier M, Arda S, Claus A, Sussmuth SD (2005). Tau protein level in cerebrospinal fluid is increased in patients with early multiple sclerosis.. Mult Scler.

[4] Semra YK, Seidi OA, Sharief MK (2002). Heightened intrathecal release of axonal cytoskeletal proteins in multiple sclerosis is associated with progressive disease and clinical disability.. J Neuroimmunol.

[5] Terzi M, Birinci A, Cetinkaya E, Onar MK (2007). Cerebrospinal fluid total tau protein levels in patients with multiple sclerosis.. Acta Neurol Scand.

[6] Brettschneider J, Petzold A, Junker A, Tumani H (2006). Axonal damage markers in the cerebrospinal fluid of patients with clinically isolated syndrome improve predicting conversion to definite multiple sclerosis.. Mult Scler.

[7] Niebroj-Dobosz I, Dziewulska D, Janik P (2006). Auto-antibodies against proteins of spinal cord cells in cerebrospinal fluid of patients with amyotrophic lateral sclerosis (ALS).. Folia Neuropathol.

[8] Silber E, Semra YK, Gregson NA, Sharief MK (2002). Patients with progressive multiple sclerosis have elevated antibodies to neurofilament subunit.. Neurology.

[9] Fialova L, Bartos A, Soukupova J, Svarcova J, Ridzon P (2009). Synergy of serum and cerebrospinal fluid antibodies against axonal cytoskeletal proteins in patients with different neurological diseases.. Folia Biol (Praha).

[10] Terryberry JW, Thor G, Peter JB (1998). Autoantibodies in neurodegenerative diseases: antigen-specific frequencies and intrathecal analysis.. Neurobiol Aging.

[11] Ehling R, Lutterotti A, Wanschitz J, Khalil M, Gneiss C (2004). Increased frequencies of serum antibodies to neurofilament light in patients with primary chronic progressive multiple sclerosis.. Mult Scler.

[12] Bartos A, Fialova L, Soukupova J, Kukal J, Malbohan I (2007). Antibodies against light neurofilaments in multiple sclerosis patients.. Acta Neurol Scand.

[13] Bartos A, Fialova L, Soukupova J, Kukal J, Malbohan I (2007). Elevated intrathecal antibodies against the medium neurofilament subunit in multiple sclerosis.. J Neurol.

[14] Svarcova J, Fialova L, Bartos A, Steinbachova M, Malbohan I (2008). Cerebrospinal fluid antibodies to tubulin are elevated in the patients with multiple sclerosis.. Eur J Neurol.

[15] Cleveland DW, Hwo SY, Kirschner MW (1977). Purification of tau, a microtubule-associated protein that induces assembly of microtubules from purified tubulin.. J Mol Biol.

[16] Binder LI, Frankfurter A, Rebhun LI (1985). The distribution of tau in the mammalian central nervous system.. J Cell Biol.

[17] Drubin DG, Kirschner MW (1986). Tau protein function in living cells.. J Cell Biol.

[18] Rosenmann H, Meiner Z, Geylis V, Abramsky O, Steinitz M (2006). Detection of circulating antibodies against tau protein in its unphosphorylated and in its neurofibrillary tangles-related phosphorylated state in Alzheimer's disease and healthy subjects.. Neurosci Lett.

[19] Lublin FD, Reingold SC (1996). Defining the clinical course of multiple sclerosis: results of an international survey. National Multiple Sclerosis Society (USA) Advisory Committee on Clinical Trials of New Agents in Multiple Sclerosis.. Neurology.

[20] McDonald WI, Compston A, Edan G, Goodkin D, Hartung HP (2001). Recommended diagnostic criteria for multiple sclerosis: guidelines from the International Panel on the diagnosis of multiple sclerosis.. Ann Neurol.

[21] Kurtzke JF (1983). Rating neurologic impairment in multiple sclerosis: an expanded disability status scale (EDSS).. Neurology.

[22] Matheus S, Deparis X, Labeau B, Lelarge J, Morvan J (2005). Discrimination between primary and secondary dengue virus infection by an immunoglobulin G avidity test using a single acute-phase serum sample.. J Clin Microbiol.

[23] Himmler A, Drechsel D, Kirschner MW, Martin DW (1989). Tau consists of a set of proteins with repeated C-terminal microtubule-binding domains and variable N-terminal domains.. Mol Cell Biol.

[24] Narita M, Yamada S, Matsuzono Y, Itakura O, Togashi T (1996). Immunoglobulin G avidity testing in serum and cerebrospinal fluid for analysis of measles virus infection.. Clin Diagn Lab Immunol.

[25] Flori P, Tardy L, Patural H, Bellete B, Varlet MN (2004). Reliability of immunoglobulin G antitoxoplasma avidity test and effects of treatment on avidity indexes of infants and pregnant women.. Clin Diagn Lab Immunol.

[26] Hedman K, Hietala J, Tiilikainen A, Hartikainen-Sorri AL, Raiha K (1989). Maturation of immunoglobulin G avidity after rubella vaccination studied by an enzyme linked immunosorbent assay (avidity-ELISA) and by haemolysis typing.. J Med Virol.

[27] Cucnik S, Kveder T, Krizaj I, Rozman B, Bozic B (2004). High avidity anti-beta 2-glycoprotein I antibodies in patients with antiphospholipid syndrome.. Ann Rheum Dis.

[28] Cui Z, Wang HY, Zhao MH (2006). Natural autoantibodies against glomerular basement membrane exist in normal human sera.. Kidney Int.

[29] Milosevic-Jovcic N, Ciric D, Hajdukovic-Dragojlovic L, Mircetic V (2004). Differences in the relationship of specificity to titre and functional affinity between circulating Ga- and pan-reactive IgM rheumatoid factors in rheumatoid arthritis.. Rheumatology (Oxford).

[30] Villalta D, Romelli PB, Savina C, Bizzaro N, Tozzoli R (2003). Anti-dsDNA antibody avidity determination by a simple reliable ELISA method for SLE diagnosis and monitoring.. Lupus.

[31] Racke MK (2008). The role of B cells in multiple sclerosis: rationale for B-cell-targeted therapies.. Curr Opin Neurol.

[32] Rosenmann H, Grigoriadis N, Karussis D, Boimel M, Touloumi O (2006). Tauopathy-like abnormalities and neurologic deficits in mice immunized with neuronal tau protein.. Arch Neurol.

[33] Graus F, Saiz A, Dalmau J (2010). Antibodies and neuronal autoimmune disorders of the CNS.. J Neurol.

[34] Zamecnik J, Cerny R, Bartos A, Jerabek J, Bojar M (2004). Paraneoplastic opsoclonus-myoclonus syndrome associated with malignant fibrous histiocytoma: neuropathological findings.. Cesk Patol.

[35] Huizinga R, Heijmans N, Schubert P, Gschmeissner S, t Hart BA (2007). Immunization with neurofilament light protein induces spastic paresis and axonal degeneration in Biozzi ABH mice.. J Neuropathol Exp Neurol.

[36] Bartos A, Pitha J (2003). Opsoclonus-myoclonus-dysequilibrium syndrome: cytological and immunological dynamics in the serial cerebrospinal fluid in two patients.. J Neurol.

